# Sarcopenia and Muscle Functions at Various Stages of Alzheimer Disease

**DOI:** 10.3389/fneur.2018.00710

**Published:** 2018-08-28

**Authors:** Yusuke Ogawa, Yoshitsugu Kaneko, Tomohiko Sato, Soichiro Shimizu, Hidekazu Kanetaka, Haruo Hanyu

**Affiliations:** Department of Geriatric Medicine, Tokyo Medical University, Tokyo, Japan

**Keywords:** Alzheimer disease, sarcopenia, dynapenia, muscle strength, muscle mass

## Abstract

Although sarcopenia is closely linked to dementia, particularly Alzheimer disease (AD), there are few studies examining the prevalence and associated factors of sarcopenia in subjects with AD. This study aimed to investigate the prevalence of sarcopenia, factors associated with sarcopenia in elderly subjects with AD, and differences in muscle functions of the upper and lower extremities and gait speed at various stages of AD. We evaluated handgrip and knee extension strength, muscle mass, and gait speed in 285 elderly outpatients with probable AD (mean age 82. 0 ± 5.3 years), including early AD (*n* = 82), mild AD (*n* = 90), and moderate AD (*n* = 113), and 67 elderly outpatients with normal cognition (NC) (mean age 81.1 ± 4.7 years). Sarcopenia was defined according to the consensus of the Asian Working Group for Sarcopenia. The prevalence rate of sarcopenia was significantly higher in early AD, mild AD, and moderate AD than in NC (11% in NC, 36% in early AD, 45% in mild AD, and 60% in moderate AD of the female group, and 13% in NC, 41% in early AD, 47% in mild AD, and 47% in moderate AD of the male group). Age, body mass index, and Mini-mental state examination score were associated with sarcopenia in female or male AD groups. Decreased muscle strength without loss of muscle mass of the upper and lower extremities in the female AD group and those of the lower extremity in the AD male group were found in early and mild stages. Both muscle strength and mass decreased in the moderate AD. Low gait speed was also found in the early female and male AD which progressed with advancing dementia. Subjects with AD, even the early stages of AD, showed a high prevalence rate of sarcopenia. Higher age, lower BMI, and lower MMSE score were associated with sarcopenia in the female or male AD. There were differences in muscle functions and physical performance between the stages of the female and male AD.

## Introduction

Sarcopenia is the degenerative loss of skeletal muscle mass and strength associated with aging and results in functional limitations, such as low gait speed (1, 2). This condition increases falls, physical impairment, poor quality of life, and mortality risk. Although the prevalence of sarcopenia varies depending on the definition or cut-off point in each study (3), the prevalence of sarcopenia in healthy adults aged ≥ 60 years accounts for 10% or more in men and women, and tends to increase with age (4). On the other hand, physical difficulties, such as decreased grip strength and low gait speed, coexist in elderly individuals with cognitive impairment and dementia. Alterations of body composition are also associated with subjects who have dementia and Alzheimer disease (AD). Burns et al. (5) found reduced lean mass in individuals with early AD and this was associated with brain atrophy. Some studies demonstrated that weight loss occurred before the diagnosis of AD (6, 7) and this was associated with the faster clinical progression of AD (8). A pathological study showed a significant negative correlation between body mass index (BMI) and AD pathology (9). Slow gait speed and decreased grip strength are associated with cognitive impairment and are related to incident cognitive decline (10, 11). These findings suggest that sarcopenia is closely linked to dementia, particularly AD, and may be involved in the pathophysiological process of AD.

However, there are few studies examining the prevalence and association of sarcopenia with cognitive impairment in elderly subjects with AD (12–14). Clarifying the relationship of both conditions may be necessary for considering interventions and appropriate care in geriatric medicine. The present study aimed to investigate (1) differences in the prevalence of sarcopenia between elderly subjects with normal cognition and those at various stages of AD, (2) relationships of muscle mass and strength of the upper and lower extremities, and gait speed with severity of dementia, and (3) factors associated with sarcopenia.

## Materials and methods

### Subjects

This study included 285 elderly outpatients with AD (102 men and 183 women, mean age 82.0 ± 5.3 years, 67–96 years old) and 67 elderly outpatients with normal cognition (NC) (30 men and 37 women, mean age 81.1 ± 4.7 years, 71–94 years old) who attended the Memory Clinic at Tokyo Medical University Hospital. The outpatients with AD had to meet the DSM-5 criteria (15) for a diagnosis of probable AD. All the outpatients underwent general physical examinations, neurological and neuropsychological examinations, laboratory tests, and brain imaging studies, such as brain CT, magnetic resonance imaging, and single photon emission CT to exclude other potential causes of dementia. The severity of cognitive impairment was assessed using the Mini-Mental State Examination (MMSE) (16). Patients with AD were divided into early AD (MMSE score ≥ 24), mild AD (MMSE score, 21–23), and moderate AD (MMSE score ≤ 20) according to the MMSE score. Subjects showing irregular and extensive periventricular hyperintensity or deep white matter hyperintensity lesions [grade 3 based on the scale of Fazekas et al. (17)] and cerebrovascular diseases, except for single lacunar infarction, were excluded. We also excluded subjects those who could not walk without assistance, and those with severe dementia (MMSE score < 12), parkinsonism, and major depression. We determined the prevalence of major chronic diseases of older adults using the Charlson comorbidity index (18). This index provides a simple and valid method of estimating risk of death from comorbid disease for use in longitudinal studies. In this study, we scored comorbid illnesses, but not age, of each subject.

We routinely assess cognitive function, and measure body composition and physical functions, including gait speed, muscle functions of the upper, and lower extremities, in almost all of our first visit subjects who have an appointment at the Memory Clinic at Tokyo Medical University. In our Memory Clinic, these measurements, including comprehensive geriatric assessment, are always used in the same way as in general physical and neurological examinations. As in this study we analyzed data obtained in clinical practice, such as cognitive and physical functions, informed consent, but not written consent, was obtained from all the subjects or their relatives. This study was approved by the ethics committee of Tokyo Medical University.

### Muscle strength and mass, and gait speed

The upper and lower muscle functions were measured using a digital handgrip meter (MCZ-5041, Macros, Tokyo, Japan) for handgrip strength and a μTas F-1 handheld dynamometer (ANIMA, Tokyo, Japan) for knee extension strength. Isometric grip strength was assessed for each hand, and the maximum grip strengths of the right and left hands were analyzed. For knee extension, we analyzed the larger value of two measurements for each leg. Inter-rater reliability of isometric knee extension strength measurements was highly correlated with the data measured by the handheld dynamometers (correlation coefficient, both men and women: *r* = 0.99) (19).

Appendicular muscle masses of the upper and lower extremities were measured using bioelectrical impedance analysis (InBody S 10, InBody Japan Inc., Tokyo, Japan). The skeletal muscle mass index (SMI) was calculated by dividing skeletal muscle mass by height (in meters) squared (kg/m^2^). Lean body mass evaluated by this device was highly correlated with the data measured by dual-energy X-ray absorptiometry (correlation coefficients: men *r* = 0.96 and women *r* = 0.95) (20).

Gait speed was assessed at usual walking pace over a distance of 6 meters in second, counted after a one meter initial distance. Gait speed measurements were performed in a flat and unobstructed clinic hallway (11).

We analyzed the data of muscle functions and gait speed in each female and male group, as muscle strength and physical performance are considerably different between women and men.

### Diagnosis of sarcopenia

Sarcopenia was defined according to the consensus of the Asian Working Group for Sarcopenia criteria (2), which include three components, namely, low handgrip strength (< 26 kg for men and < 18 kg for women), low gait speed (≤0.8 m/s), and low muscle mass as assessed using SMI (7.0 kg/m^2^ for men and 5.7 kg/m^2^ for women as measured by bioelectrical impedance analysis).

### Statistical analysis

Values were expressed as mean ± SD. Statistical analysis was performed using the Student's *t* test, χ^2^ test, and one-way analysis of variance with the *post-hoc Fisher's* partial least-squares difference test. Factors associated with sarcopenia were analyzed using multivariate logistic regression analysis. A *p*-value < 0.05 was considered to indicate a statistically significant difference.

## Results

### Clinical characteristics and findings of the NC, early AD, mild AD, and moderate AD in the female group (Table 1)

There were no significant differences in age among the groups. Education was significantly lower in the mild AD and moderate AD groups than in the NC group, and in the moderate AD group than in the early AD group. As the AD groups were divided according to the MMSE score, there were significant differences in MMSE scores between the groups. The Charlson comorbidity indices were significantly higher in the early AD, mild AD, and moderate AD groups than in the NC group because 1 point is added in each individual with dementia, and were significantly higher in the mild AD and moderate AD groups than in the early AD group. BMI was significantly lower in the moderate AD group than in the mild AD group. SMI was significantly lower in the moderate AD group than in the NC, early AD, and mild AD groups. For the upper extremity, the handgrip strength was significantly lower in the early AD, mild AD, and moderate AD groups than in the NC group, and in the moderate AD group than in the early AD group, but the arm muscle mass was significantly lower only in the moderate AD group than in the NC, early AD, and mild AD groups. For the lower extremity, the leg strength was significantly lower in the early AD, mild AD, and moderate AD groups than in the NC group, and in the moderate AD group than in the mild AD group, but the leg muscle mass was significantly lower only in the moderate AD group than in the NC, early AD, and mild AD groups. Gait speed was significantly lower in the early AD, mild AD, and moderate AD groups than in the NC group, and in the moderate AD group than in the early AD group. The prevalence rate of sarcopenia was significantly higher in the early AD, mild AD, and moderate AD groups than in the NC group, and in the moderate AD group than in early AD group.

**Table 1:** Clinical characteristics and findings of the NC, early AD, mild AD and mod. AD in the female group.

	**NC**	**Early AD**	**Mild AD**	**Mod. AD**	**Differences (*p*-value)**
No. of subjects	37	50	58	75	
Age (years)	81.1 ± 4.8	81.2 ± 5.1	82.9 ± 5.2	82.6 ± 5.9	
Educatrion (years)	12.6 ± 2.5	12.4 ± 2.3	11.6 ± 2.4	11.3 ± 2.2	NC > Mo.d AD (^*^), Mod. AD (^**^), Early AD >Mod. AD (^*^)
MMSE score	27.3 ± 2.3	25.0 ± 1.0	22.0 ± 0.9	17.4 ± 2.4	NC > Early AD (^****^) > Mild AD (^****^) > Mod. AD (^****^)
Charlson comorbidity index	0.47 ± 0.51	1.48 ± 0.61	1.79 ± 0.67	1.71 ± 0.63	NC > Early AD (^****^), Mild AD (^****^), Mod. AD (^****^) Early AD < Mild AD (^*^), Mod. AD (^*^)
BMI (kg/m^2^)	22.8 ± 3.0	22.6 ± 2.6	22.8 ± 3.6	21.5 ± 3.7	Mild AD > Mod. AD (^*^)
SMI (kg/m^2)^	5.76 ± 0.73	5.69 ± 0.58	5.62 ± 0.82	5.26 ± 0.65	NC (^***^), Early AD (^**^), Mild AD (^**^) > Mod. AD
**UPPER EXTREMITY**					
Handgrip strength (kg)	20.1 ± 3.3	17.4 ± 3.7	16.9 ± 3.7	15.8 ± 3.8	NC (^**^) > Early AD (^**^), Mild AD (^****^), Mod. AD (^****^) Early AD > Mod. AD (^*^)
Arm muscle mass (kg)	1.47 ± 0.24	1.47 ± 0.24	1.42 ± 0.33	1.28 ± 0.26	NC (^***^), Early AD (^***^), Mild AD (^**^) > Mod. AD
**LOWER EXTREMITY**					
Leg strength (kg)	21.7 ± 4.7	17.3 ± 4.9	16.8 ± 5.1	15.1 ± 5.3	NC (^**^) > Early AD (^**^), Mild AD (^****^), Mod. AD (^****^) Early AD > Mod. AD (^*^)
Leg muscle mass (kg)	5.00 ± 0.74	4.92 ± 0.79	4.72 ± 0.87	4.47 ± 0.65	NC (^***^), Early AD (^**^), Mild AD (^*^) > Mod. AD
Gait speed (m/s)	1.08 ± 0.18	0.97 ± 0.19	0.93 ± 0.18	0.90 ± 0.19	NC > Early AD (^*^), Mild AD (^***^), Mod. AD (^****^) Early AD > Mod. AD (^**^)
Prevalence of sacropenia (%)	4 (11%)	18 (36%)	26 (45%)	45 (60%)	NC < Early AD (^*^), Mild AD (^**^), Mod. AD (^****^) Early AD > Mod. AD (^*^)

NC: Normal cognition, AD: Alzheimer disease, Mod. moderate, MMSE: Mini-mental state examination; BMI: Body mass index, SMI: Skeletal muscle mass index

* *p < 0.05*,

** *p < 0.01*,

*** *p < 0.001*,

**** *p < 0.0001*.

### Clinical characteristics and findings of the NC, early AD, mild AD, and moderate AD in the male group (Table 2)

There was no significant difference in the age among the groups. Education was significantly lower in the mild AD group than in the NC group. Similar to the female group, there were significant differences in MMSE scores between the groups. The Charlson comorbidity indices were significantly higher in the early AD, mild AD, and moderate AD groups than in the NC group. Although BMI was similar among the groups, SMI was significantly lower in the moderate AD group than in the NC group. For the upper extremity, there were no significant differences in the handgrip strength and arm muscle mass among the groups. For the lower extremity, the leg strength was significantly lower in the early AD, mild AD, and moderate AD groups than in the NC group, but the leg muscle mass was significantly lower in the moderate AD group than in the NC group. Gait speed was significantly lower in the early AD, mild AD, and moderate AD groups than in the NC group. The prevalence rate of sarcopenia was significantly higher in the early AD, mild AD, and moderate AD groups than in the NC group.

**Table 2:** Clinical characteristics and findings of the NC, early AD, mild AD and mod. AD in the male group.

	**NC**	**Early AD**	**Mild AD**	**Mod. AD**	**Differences (*p*-value)**
No. of subjects	30	32	32	38	
Age (years)	80.1 ± 4.4	82.3 ± 4.0	81.9 ± 4.7	81.3 ± 6.5	
Educatrion (years)	13.3 ± 2.7	13.8 ± 2.8	14.9 ± 1.8	14.4 ± 2.3	NC > Mild AD (^*^)
MMSE score	27.5 ± 1.4	24.4 ± 1.0	22.1 ± 0.7	18.0 ± 2.3	NC > Early AD (^****^) > Mild AD (^****^) > Mod AD (^****^)
Charlson comorbidity index	0.86 ± 0.64	1.69 ± 0.64	1.78 ± 0.66	1.74 ± 0.76	NC < Early AD (^****^), Mild AD (^****^), Mod. AD (^****^)
BMI (kg/m^2^)	23.5 ± 2.2	23.5 ± 2.8	23.3 ± 2.3	22.7 ± 3.6	
SMI (kg/m^2)^	7.12 ± 0.64	6.97 ± 0.61	7.02 ± 0.77	6.75 ± 0.81	NC > Mod. AD (^*^)
**UPPER EXTREMITY**					
Handgrip strength (kg)	27.0 ± 4.8	25.5 ± 4.9	24.5 ± 6.4	24.8 ± 5.3	
Arm muscle mass (kg)	2.23 ± 0.36	2.17 ± 0.30	2.14 ± 0.30	2.08 ± 0.43	
**LOWER EXTREMITY**					
Leg strength (kg)	29.3 ± 7.6	24.8 ± 4.2	24.4 ± 9.9	22.4 ± 8.2	NC > Early AD (^*^), Mild AD (^*^), Mod. AD (^***^)
Leg muscle mass (kg)	7.19 ± 0.94	7.08 ± 0.94	7.05 ± 0.92	6.70 ± 1.01	NC > Mod. AD (^*^)
Gait speed (m/s)	1.15 ± 0.25	1.01 ± 0.26	0.95 ± 0.19	0.93 ± 0.16	NC > Early AD (^*^), Mild AD (^***^), Mod. AD (^***^)
Prevalence of sacropenia (%)	4 (13%)	13 (41%)	15 (47%)	18 (47%)	NC < Early AD (^*^), Mild AD (^**^), Mod. AD(^**^)

NC: Normal cognition, AD: Alzheimer disease, Mod. moderate, MMSE: Mini-mental state examination; BMI: Body mass index, SMI: Skeletal muscle mass index

* *p < 0.05*,

** *p < 0.01*,

*** *p < 0.001*,

**** *p < 0.0001*.

### Pattern diagram of parameters at various stages of AD compared with the NC

**Figure 1:**
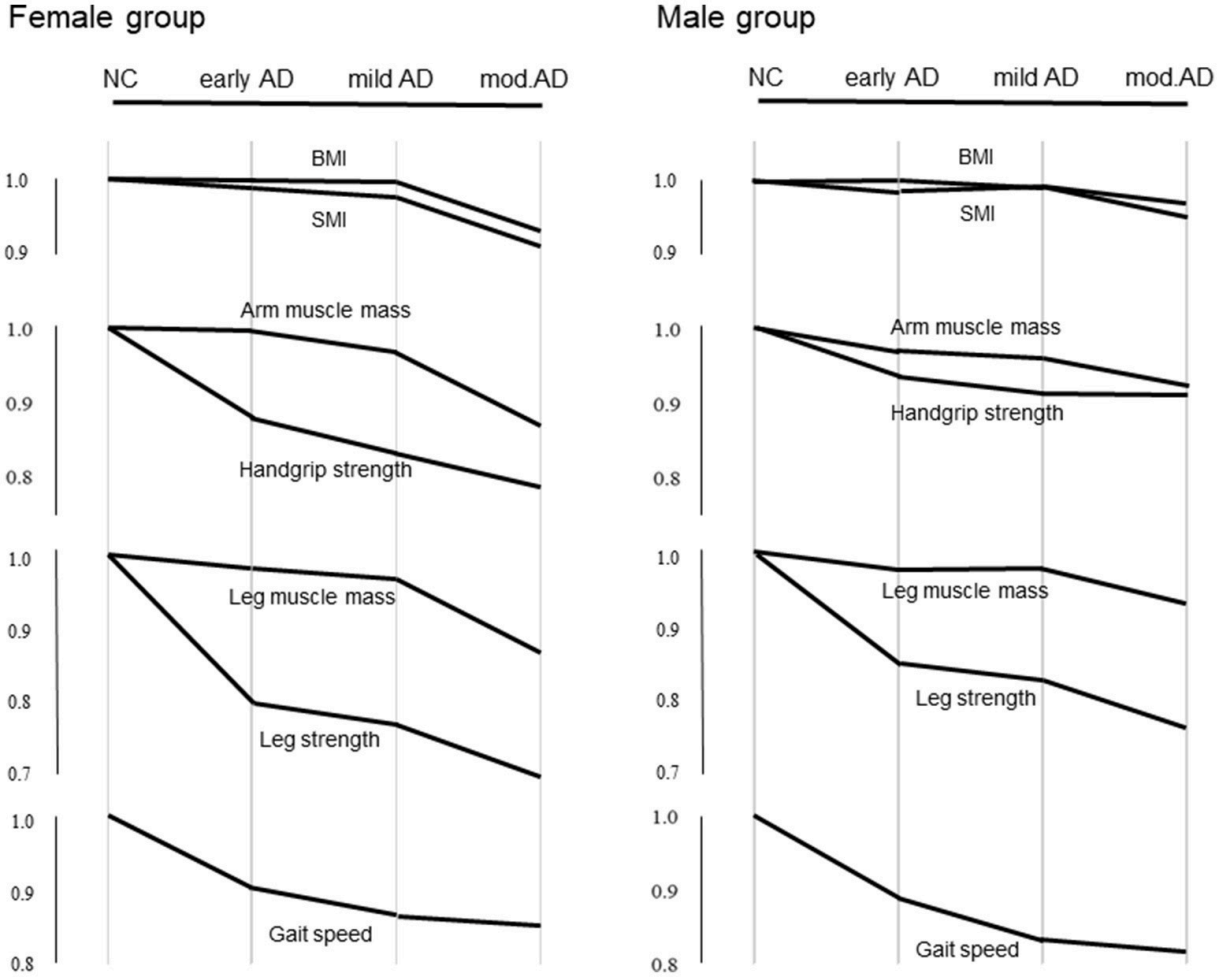
Differences in average values of BMI, SMI, muscle strengths and masses in the upper and lower extremities, and gait speed at various stages of AD compared with the NC. The Y axis indicates reduction from the mean values of the parameters in the NC group. Values in early, mild, and moderate AD groups are normalized to the mean of the NC group.

Differences in the average values of BMI, SMI, muscle strengths, and masses in the upper and lower extremities, and gait speed at various stages of AD compared with the NC group are shown in Figure 1.

### Factors associated with sarcopenia in the female and male AD groups (Table 3)

Variables, including age, education, BMI, MMSE, and Charlson comorbity index, were entered on stepwise logistic analysis. Factors associated with sarcopenia in the female and male AD groups are shown in Table 3. Stepwise logistic regression analysis showed that age (*p* < 0.001, OR = 1.16), BMI (*p* < 0.001, OR = 0.76), and MMSE score (*p* < 0.05, OR = 0.89) were associated with sarcopenia in the female AD group, and shows that age (*p* < 0.01, OR = 1.17) and BMI (p < 0.01, OR = 0.76) were associated with sarcopenia in the male AD group.

**Table 3:** Factors associated with sarcopenia as determined by stepwise logistic regression analysis.

**Variable**	**OR**	**95%Cl**	***p*-value**
**FEMALE GROUP**			
Age	1.16	1.08–1.24	<0.001
BMI	0.76	0.68–0.86	<0.001
MMSE	0.89	0.81–0.99	<0.05
**MALE GROUP**			
Age	1.17	1.07–1.29	<0.01
BMI	0.76	0.64–0.90	<0.01

*Variables entered on stepwise logistic regression analysis: age, education, BMI: Body Mass Index, MMSE: Mini-mental state examination, and Charlson comorbidity index*.

## Discussion

We found that the prevalence rate of sarcopenia was significantly higher in the early AD, mild AD, and moderate AD than in the NC in the female group (11% in NC, 36% in early AD, 45% in mild AD, and 60% in moderate AD) and in the male group (13% in NC, 41% in early AD, 47% in mild AD, and 47% in moderate AD). Age, BMI, and MMSE score were associated with sarcopenia among female or male subjects with AD. Decreased muscle strength without loss of muscle mass in the upper and lower extremities of the female group and those in the lower extremity of the male group were found in the early and mild AD, and both muscle strength and muscle mass decreased in the moderate AD. Low gait speed was also found in the early stage of AD and progressed with advancing dementia. Our results suggest that decreased muscle strength without loss of muscle mass in the upper or lower extremities and low gait speed are early non-cognitive features of elderly patients with AD. In addition, the alterations of muscle functions in the upper and lower extremities differed between the stages of female and male AD.

There are few studies regarding the prevalence rate of sarcopenia in subjects with AD (12–14). Sugimoto et al. (12) described that the prevalence rate of sarcopenia was 8.6% in NC, 12.5% in amnestic MCI, and 23.3% in AD. We found a high prevalence rate of sarcopenia in both the NC and AD groups. Although the prevalence rate of sarcopenia may be dependent on definitions (European Working Group on sarcopenia in Older People vs. Asian Working Group for sarcopenia) or cut-off values, the different ages in each study (mean age, 77 years vs. 82 years) mainly explains the different prevalence rates. Indeed, the prevalence rate of sarcopenia in community-dwelling Japanese older adults (aged 65 to 89 years) was reported to be about 20% (21). However, consistent with their results, we also found that subjects with AD, even at the early stage, showed a higher prevalence rate of sarcopenia than those with NC.

There are common underlying conditions shared by sarcopenia and AD, such as inflammation, oxidative stress, nutrition, immobility, and hormonal dysregulation (22–24). Notably, a significant correlation between lean mass and whole brain volume was observed in subjects with AD (5). A recent positron emission tomography study revealed an association between amyloid-β deposits in the brain and low gait speed in healthy elderly individuals and subjects with MCI (25). A decreased SMI in subjects with AD was reported to be associated with clinical dementia rating (CDR) (13). In addition to age and BMI, our findings support a relationship between sarcopenia and severity of dementia as assessed using MMSE in the female AD group. Chronic conditions, such as diabetes, hypertension, cerebrovascular disease, chronic obstructive pulmonary disease, heart failure, and osteoporosis, are associated with sarcopenia (3). Although we found no significant relationship between sarcopenia and Charlson comorbidity index, correlation of each comorbid disease with sarcopenia should be investigated. Indeed, when analyzing all subjects including the female and male AD groups, a significant relationship between Charlson comorbidity index and sarcopenia was found. Therefore, the possibility that some chronic diseases are associated with sarcopenia cannot be ruled out. Some researchers have revealed that a low serum level of 25-hydroxyvitamin D is associated with sarcopenia (3, 12), blood biomarkers for sarcopenia have not yet been identified. The mechanisms causing low muscle strength and mass involve multifactorial and complex processes.

Reductions in muscle strength of the upper and lower extremities were relatively higher than those in muscle mass in each stage of the AD group. We found a decreased muscle strength without loss of muscle mass of the upper or lower extremities in subjects with early AD and mild AD. This condition may be explained by the decreased muscle quality, or may be termed as dynapenia (age-related loss of muscle strength and power) (26, 27). Although pathophysiological mechanisms of poor muscle quality remain unclear, muscle quality usually decreased with aging (28), and was observed in older individuals with dementia or diabetes (29, 30). Indeed, when muscle quality was expressed as the ratio of muscle strength to muscle mass, such as grip strength/arm skeletal muscle mass and knee extension strength/leg skeletal muscle mass, similar to other reports in the literature (28–30), these ratios in the upper and lower extremities were significantly lower in the female AD group than the female NC group and those in the lower extremity were significantly lower in the male AD group than the male NC group. In particular, prominently decreased muscle quality was found even in the early AD. The pathophysiology of dynapenia may be different from that of sarcopenia. In dynapenia, muscle strength is lost at a substantially faster rate than muscle mass. In addition, loss of muscle strength is a more consistent risk for physical disability than loss of muscle mass (31). Therefore, potential therapy and rehabilitation strategies in dynapenia may be different from those in sarcopenia. It remains uncertain why there are differences in muscle functions between various stages of AD. In the male AD group, no significant differences in handgrip strength and arm muscle mass were found between the NC group and various stages of AD. These results suggest that measurements of muscle functions in the lower extremity may be more sensitive for detecting physical disability than those in the upper extremity in the male AD. In addition to sex hormones, nutritional status, physical activity, comorbidities, and other intrinsic and extrinsic factors may be associated with sex-related muscle functions (32). Further studies are needed to clarify sex-related skeletal muscle metabolism.

Our study had some limitations. First, because this study used a cross-sectional design, a longitudinal study is needed to confirm our results. Second, we excluded subjects with MCI, because MCI is a heterogenous condition and underlies various neuropathologies, besides AD. Indeed, a recent large autopsy study demonstrated that less than one-quarter of MCI subjects showed pure AD pathology (33). Our aim in this study was to investigate the relationship between muscle functions and severity of dementia at various stages of AD. In addition, further studies are needed to determine the relationships between sarcopenia and other types of dementia, such as vascular dementia and dementia with Lewy bodies, besides AD. Third, the stage of AD was determined using MMSE but not CDR because of the lack of data. Although MMSE can be used as a surrogate measure for the CDR for the staging of dementia in AD (34), each cut-off point of the MMSE score differentiating early AD, mild AD, and moderate AD in this study may be arbitrary. We believe that most subjects of the early AD group (MMSE score ≥ 24) in this study corresponds to CDR global score = 0.5. Fourth, although we used Charlson comorbidity index in the assessment for comorbid conditions of individuals, the severity and duration of comorbidities may also be associated with sarcopenia. Finally, we consider that body compositions and muscle functions may be affected by season and outside temperature.

## Conclusion

Although muscle functions and physical performance decrease with aging, these functions further decrease in the early stage of AD. Subjects with AD, even the early stages of AD, showed a high prevalence rate of sarcopenia. Higher age, lower BMI, and lower MMSE score were associated with sarcopenia in the female or male AD. There were differences in muscle functions and physical performance between the stages of the female and male AD. Decreased muscle strength without loss of muscle mass in the upper or lower extremities and low gait speed may be early non-cognitive features of elderly subjects with AD. It may be necessary to measure the muscle functions of not only the upper extremity but also the lower extremity in elderly subjects with AD to accurately detect physical disability.

## Ethics statement

All procedures performed in the studies involving human participants were in accordance with the ethical standards of the institutional or national research committee and with 1964 Helsinki declaration and its later amendments or comparable ethical standards. As in this study we analyzed data obtained in clinical practice, such as cognitive and physical functions, informed consent, but not written consent, was obtained from all the subjects or their relatives.

## Author contributions

YO, YK, and HH designed the study. TS, SS, and HK collected the data and performed clinical assessment. YO wrote the manuscript, and HH revised the manuscript.

### Conflict of interest statement

The authors declare that the research was conducted in the absence of any commercial or financial relationships that could be construed as a potential conflict of interest. The reviewer RK and handling Editor declared their shared affiliation.
